# The context dependency of fish‐habitat associations in separated karst ecoregions

**DOI:** 10.1002/ece3.10701

**Published:** 2023-12-18

**Authors:** Dusty A. Swedberg, Robert Mollenhauer, Shannon K. Brewer

**Affiliations:** ^1^ Oklahoma Cooperative Fish and Wildlife Research Unit Oklahoma State University Stillwater Oklahoma USA; ^2^ U.S. Geological Survey, Oklahoma Cooperative Fish and Wildlife Research Unit Oklahoma State University Stillwater Oklahoma USA; ^3^ Present address: Prairie Research Institute, Illinois Natural History Survey Champaign Illinois USA; ^4^ Present address: Heart of the Hills Fisheries Science Center Texas Parks and Wildlife Department Mountain Home Texas USA; ^5^ Present address: U.S. Geological Survey, Alabama Cooperative Fish and Wildlife Research Unit, 203 Swingle Hall Auburn University Auburn Alabama USA

**Keywords:** conservation, detection, groundwater, multiscale habitat, occupancy models, spring‐associated, temperature

## Abstract

Fish populations may be isolated via natural conditions in geographically separated ecoregions. Although reconnecting these populations is not a management goal, we need to understand how these populations persist across landscapes to develop meaningful conservation actions, particularly for species occupying sensitive karst ecosystems. Our study objective was to determine the physicochemical factors related to the occurrence of four spring‐associated fishes. Arbuckle Uplift and Ozark Highlands ecoregions, USA. We used a hierarchical approach to identify habitat relationships at multiple spatial scales. We collected detection data using snorkeling and seining. We examined the physicochemical relationships related to the detection and occurrence of four spring‐associated fishes using occupancy modeling in a Bayesian framework. We found physicochemical relationships that differed and were similar between ecoregions for several fishes. For three species, we found different water temperature relationships between ecoregions. Smallmouth bass were ubiquitous in their use of drainage areas in the Ozark Highlands but only associated with the lower network of the Arbuckle Uplift. There were several mirrored relationships between ecoregions, including an interaction between residual pool depth and water temperature, where sites with deeper pools were more likely to be occupied during warmer water temperatures. There were single‐species occurrence relationships with percent vegetation and percent agriculture. Lastly, snorkeling was a more efficient sampling method compared to seining for all fishes. Our results indicate stream temperature mitigation may be possible via the maintenance of key channel morphologies, and we identify shared stressors between ecoregions. Channel mitigation to maintain reaches with deeper pools may be an important strategy for maintaining thermal refugia, particularly when considering climate change. Identifying the mechanistic underpinning of other multiscale ecological relationships would be helpful to discern if some of the different ecoregion relationships represent warning signals or interactions with unmeasured biotic or abiotic factors.

## Graphical abstract

Fish populations may be isolated via natural conditions in geographically separated ecoregions. Although reconnecting these populations is not a management goal, we do need to understand how these populations persist across different landscapes to develop meaningful conservation actions, particularly for species occupying sensitive karst ecosystems. Our study objective was to determine the physicochemical factors related to the occurrence of four spring‐associated fishes.

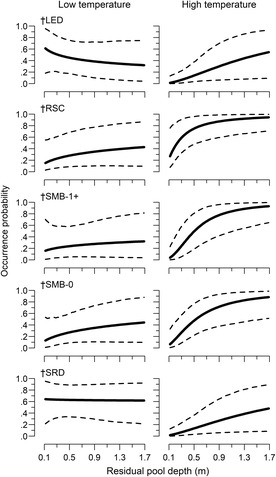

## 1 INTRODUCTION

Populations within broader species distributions can become isolated by natural phenomena; thus, their distributions may be related to different physicochemical conditions depending on the surrounding landscape. Natural features that isolate populations include glacial movements (Berendzen et al., 2010), stream capture (Buth & Mayden, 1981), and tectonic lifts (McKeown et al., 1988). For example, many fishes in the Ozark Highlands and Great Plains ecoregions appear to have recolonized northern areas of the range from a southern Ozark Highland refuge (e.g., carmine shiner *Notropis percobromus*, Berendzen et al., 2008). Distribution information on populations is useful for a variety of conservation and management needs including identifying habitat refugia over time (Lake, 2000; Magoulick & Kobza, 2003; Peterson & Rabeni, 1996; Torgersen et al., 1999), identifying locations to manage with limited resources (Dauwalter & Rahel, 2008; Gardner et al., 2013; Gore et al., 2001; Park et al., 2003; Rabeni & Sowa, 1996; Wilson et al., 2005), determining species conservation status (e.g., goldline darter *Percina aurolineata*, Albanese et al., 2004; Potoka et al., 2016), and identifying areas of reintroduction (Bearlin et al., 2002; Wall et al., 2004). Reconnecting naturally isolated populations is not a management goal; however, understanding how these populations persist across different landscapes is helpful to developing meaningful conservation and management actions (i.e., are the populations limited by different physicochemical conditions?).

The distribution of stream fauna is related to physicochemical relationships at multiple spatial scales (Hynes, 1975; Poff et al., 1997; Vannote et al., 1980). Coarse‐scale distributions of stream fishes are constrained primarily by factors that change relatively slowly such as climate and geology (Hynes, 1975; Marsh‐Matthews & Matthews, 2000). However, a myriad of physicochemical factors determines stream fish distributions at finer spatial scales (e.g., segments, reaches, and microhabitats, Goldstein & Meador, 2004; Poff et al., 1997; Southwood, 1977; Vannote et al., 1980). For example, groundwater (Brewer, 2013a; Power et al., 1999) and water temperature (Constantz, 1998; Last et al., 2011; Wehrly et al., 2006; Wolf et al., 2019) affect habitat selection by stream fishes at different spatial scales (e.g., patch or reach) and seasonally. Viewing stream fauna and their relationships with physicochemical factors at a single scale can lead to erroneous conclusions (Dunham & Vinyard, 1997). For example, pool habitat is important to smallmouth bass *Micropterus dolomieu* at a fine scale (Brewer, 2013b), but the abundance of smallmouth bass declines when pool habitat area increases at the reach scale (Brewer, 2013b). A more insightful approach is to view distributions in a hierarchical framework (Frissell et al., 1986). By viewing physicochemical relationships with fish presence in a hierarchical framework, coarse‐scale relationships can help guide conservation practices across the riverscape (Fausch et al., 2002; Rabeni & Sowa, 1996).

Groundwater creates suitable habitat patches for some species across multiple spatial scales (Brewer, 2013a). Stream reaches influenced by groundwater are important determinants of a stream's thermal regime (Caissie, 2006). Groundwater can create important thermal habitats for stream organisms (Caissie, 2006; Farless & Brewer, 2017; Glazier, 1991; Hubbs, 1995). These habitats often have different water chemistry, temperature, and ecological structure and function within the stream network (Hubbs, 1995). Spring‐associated species tend to be emblematic of karst regions (Bergey et al., 2008; Hubbs, 2001; Matthews et al., 1985); however, these species also occupy narrow spatial extents and are typically understudied (Bergey et al., 2008; Kollaus & Bonner, 2012; Matthews et al., 1985; Spitale, 2012). Groundwater species with narrow distributions (e.g., watercress darter *Etheostoma nuchale*, Duncan et al., 2010) or patchy distributions, (e.g., Arkansas darter *Etheostoma cragini*, Groce et al., 2012) often occupy locations with above‐average stream quality and provide areas of focus for conservation and management (Fausch et al., 1990). Spring‐associated species play an important ecological role as they are often the primary consumers of invertebrates (e.g., herbivorous insects, Cordes & Page, 1980) and reside in locations where insectivorous fishes are functionally very important (i.e., few piscivores, Bergey et al., 2008; Matthews et al., 1985). Although there is a general consensus that spring systems are important for a variety of biota and human ecosystem services (Biggs et al., 2017), we do not have a good understanding of the habitat needs of organisms that are endemic to springs in different ecoregions.

Our study objective was to identify the multiscale factors related to variation in occurrence among habitat patches for four spring‐associated fishes that occupy two separated ecoregions: least darter *Etheostoma microperca*, redspot chub *Nocomis asper*, southern redbelly dace *Chrosomus erythogaster*, age 1+ smallmouth bass and age‐0 smallmouth bass (≤85 mm total length). We examined differences in occurrence probability both across and between ecoregions. Our focal species represent emblematic assemblage members that are associated with higher groundwater flow (Brewer, 2013a; Seilheimer & Fisher, 2010). These four species have been documented to be associated with groundwater‐fed reaches in both study ecoregions. Additionally, the populations in eastern Oklahoma are the southern‐most extent of the species' native ranges where water temperature can be more important to their persistence. The critical thermal maximum of juvenile smallmouth bass is ~35°C (adult fishes; Brewer et al., 2022) and non‐spring‐fed streams in these ecoregions commonly exceed this threshold. Species associated with higher groundwater flows are more susceptible to disturbances because of the limited available habitat. Groundwater sources can easily become polluted or extracted to the point where they no longer create thermally unique patches (Matthews et al., 1985). Smallmouth bass is also the top‐level predator in smaller streams and these streams are important habitats for age‐0 survival (Miller & Brewer, 2021) and growth in our study ecoregions (Brewer, 2013a; Brewer & Orth, 2014).

## 2 METHODS

### 2.1 Study area

**FIGURE 1:**
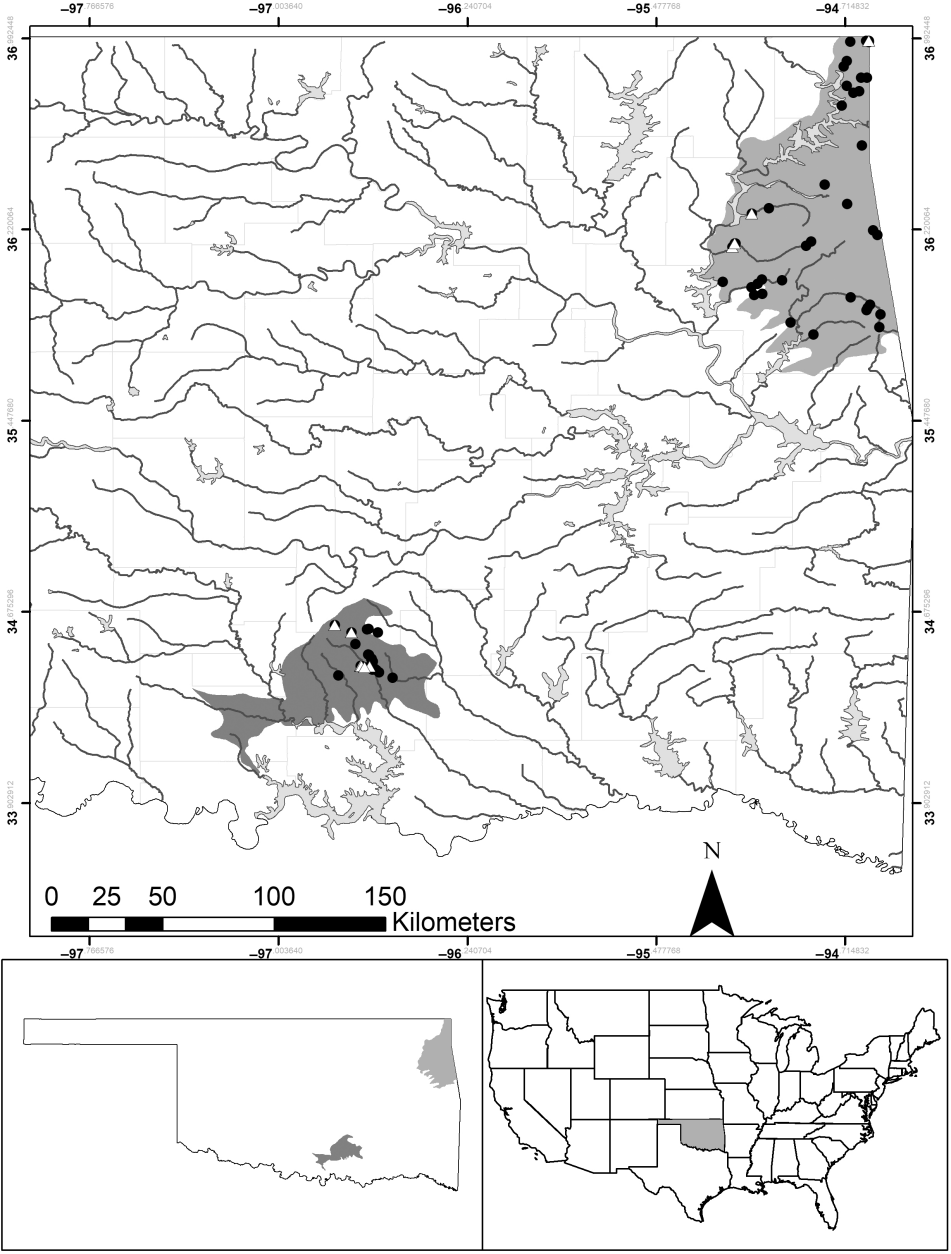
Reaches sampled (black circles) in summer 2018 (30 reaches) and 2019 (31 reaches) and least darter detections (black triangles) in the Arbuckle Uplift ecoregion (dark gray, Woods et al., 2005) and Ozark Highland ecoregion (light gray, Woods et al., 2005) of Oklahoma. This figure was produced using ArcGIS (10.7.1, Redlands, CA). Data layers for ecoregions (level 3 and level 4 ecoregions United States Environmental Protection Agency, https://www.epa.gov/eco‐research/level‐iii‐and‐iv‐ecoregions‐continental‐united‐states); state boundaries and counties (U.S. Department of Agriculture, Geospatial data gateway, https://datagateway.nrcs.usda.gov/GDGOrder.aspx?order=QuickState); and National Hydrography Data Plus (U.S. Environmental protection Agency, https://www.epa.gov/waterdata/get‐nhdplus‐national‐hydrography‐dataset‐plus‐data) are publicly available.

We sampled fishes and instream habitats in both the Ozark Highlands (level‐3, United States Environmental Protection Agency, 2017) and Arbuckle Uplift (level‐4, United States Environmental Protection Agency, 2017) ecoregions (hereafter ecoregion, Figure 1). The Ozark Highlands ecoregion encompasses portions of northeast Oklahoma, southern Missouri, southeast Kansas, and northern Arkansas, USA. The ecoregion is relatively humid (102–122 cm precipitation annually, Woods et al., 2005), limestone dominated, and comprises mixed deciduous forest with lowland grassland and pasture areas (Woods et al., 2005). The Arbuckle Uplift rises in south‐central Oklahoma and the ecoregion receives 96–109 cm of precipitation annually. The Arbuckle Uplift is dominated by dolostone, limestone, and granite lithologies (Woods et al., 2005). Land cover comprises tallgrass prairie and oak savannas, with cropland and pasture occurring in lowland areas (Woods et al., 2005). Both ecoregions are influenced by karst topography (i.e., dissolved carbonate terrains) and spring flow characteristics (Woods et al., 2005). Springs of the Ozark Highlands vary substantially in size (Vineyard & Feder, 1982) and discharge within the stream channel or contribute to surface waters via hyporheic flow (Zhou et al., 2018). Springs of the Arbuckle Uplift ecoregion are typically isothermic (Christenson et al., 2011; Osborn, 2009) and located on small spring branches. Threats to riverine biota in both ecoregions include impoundments, land‐use practices including mining and irrigation (Christenson et al., 2011), poultry pollution (Olsen et al., 2012), altered flow patterns, degraded water quality, and accelerated streambank erosion (Woods et al., 2005).

### 2.2 Fish sampling

At each riffle‐run‐pool complex (hereafter site, where a riffle was classified by higher gradient and a run as a transitional area between depositional pools and riffles, Rabeni & Jacobson, 1993), two temporally replicated surveys were conducted to account for imperfect gear detection (MacKenzie et al., 2002; Tyre et al., 2003). Because spring‐associated species can be patchily distributed but also locally abundant (Pflieger, 1997), we anticipated an average detection probability of .50 (the species was equally likely to be observed as not observed) when designing our study.

We used both snorkeling and seining to complete surveys during the baseflow, summer period (July–August) over 2 years (2018–2019). Two sampling approaches provided more flexibility because neither method was ideal at all sites (i.e., some were too shallow to snorkel or too deep to seine). Access locations were identified based on permission from private landowners and locations adjacent to streams. Sites (typically 100–200 m in length) were selected randomly on lands where we had access permission and sampled twice during the season, resulting in four surveys at sites where both methods were possible, to ensure heterogeneity in the detection probability estimates (Dunham et al., 2009; MacKenzie et al., 2002). We snorkeled prior to seining to prevent altering the habitat because snorkeling is minimally invasive. Snorkel surveys followed the general standardized protocol described by Dunham et al. (2009) and were conducted between 0800 hours and 1800 hours (i.e., when daylight was most conducive to sampling, Spyker & Van Den Berghe, 1995) at sites with horizontal visibility >1 m. We snorkeled all wadeable habitat in an upstream direction at approximately 2 m/min. Snorkelers walked slowly upstream and visually scanned the streambed in areas too shallow to submerge their mask completely. Depending on channel width, we used either one or two persons where snorkelers were randomly assigned to a lane (Thurow, 1994). We seined using the standardized protocol described by Rabeni et al. (2009) and used multiple seine hauls (1.5 m × 3.0 m sein with 1.5 mm mesh) to cover all applicable stream habitat.

### 2.3 Physicochemical conditions

We measured covariates at each site (i.e., riffle‐run‐pool complex) hypothesized to relate to stream fishes' detection (Table 1). Mean water‐column velocity (0.6 of water depth at depths <1.0 m, Gordon et al., 2004) and average water depth of each site were measured along three, evenly spaced transects perpendicular to streamflow using a Marsh McBirney Flo‐mate (Hach, Loveland, Colorado) and wading rod. Coarse substrate was estimated as a percent of the available substrate ≥90 mm diameter (Wentworth, 1922). Percent coverage of coarse wood (1.0 m^2^, i.e., circumference > 10 cm, Dodd et al., 2008) and emergent vegetation were visually estimated at each site. Because water clarity is related to fish detection (Thurow, 1994), we measured horizontal clarity using a Secchi disk. A single value was applied to multiple sites if they occurred within the same reach because we did not observe differences in water clarity between nested sites.

**TABLE 1:** Detection and occurrence of covariate summary statistics hypothesized to relate to the detection and occurrence of fish in streams.

Covariate	Arbuckle uplift mean ± SD (range)	Ozark highlands mean ± SD (range)
Detection		
Coarse wood (%)	14.00 ± 13.18 (0.00–65.00)	16.00 ± 16.51 (0.00–75.00)
Coarse substrate (%)	25.00 ± 18.71 (0.00–70.00)	28.00 ± 20.40 (5.00–85.00)
Vegetation (%)	25.00 ± 29.17 (0.00–95.00)	14.00 ± 18.60 (0.00–95.00)
Water velocity (m/s)	0.17 ± 0.11 (0.00–0.47)	0.16 ± 0.13 (0.00–0.64)
Water depth (m)	0.30 ± 0.16 (0.09–0.97)	0.27 ± 0.14 (0.06–0.72)
Water clarity (m)^a^	2.70 ± 2.08 (0.20–11.50)	4.40 ± 1.61 (1.60–11.20)
Water temperature (°C)^a^	22.96 ± 3.87 (14.20–30.80)	23.84 ± 2.73 (17.00–8.80)
Occurrence		
Fine substrate (%)	39.00 ± 26.65 (0.00–90.00)	10.00 ± 11.78 (0.00–85.00)
Residual pool depth (m)	0.54 ± 0.40 (0.00–1.95)	0.74 ± 0.53 (0.02–2.20)
Seepage run (m/s^3^)	0.02 ± 0.09 (−0.14–0.45)	0.03 ± 0.15 (−0.24–1.26)
Water temperature (°C)^a^	23.40 ± 3.56 (17.07–28.85)	23.15 ± 2.52 (16.14–27.77)
Drainage area (km^2^)^a^	73.65 ± 110.25 (1.00–329.08)	92.56 ± 94.9 (15.82–543.90)
Agriculture (%)^a^	18.31 ± 8.75 (0.01–33.57)	43.30 ± 17.14 (5.70–74.18)
Pool (%)	59.00 ± 24.00 (0.00–98.00)	0.54 ± 0.26 (0.00–96.00)
Vegetation (%)	25.00 ± 29.39 (0.00–93.00)	14.73 ± 18.63 (0.00–90.00)
Coarse wood (%)	14.00 ± 12.56 (0.00–52.00)	15.67 ± 16.13 (0.00–70.00)

Abbreviation: SD, Standard deviation.

^a^ Reach scale.

We quantified both site and reach covariates to determine the multiscale factors associated with species occurrence. First, the surface area (1.0 m^2^) of each channel unit (i.e., pool, riffle, and run) at each site was estimated by averaging wetted width at approximately three points and multiplying by the channel unit length. These values were summed for the total area of a site (m^2^). Additionally, we visually estimated the percent of sand and silt at each site because the least darter has been associated with finer substrates (Burr & Page, 1979). Percent coarse wood, percent vegetation, and average site velocity were quantified as previously described for detection. Residual pool depth (RPD) of each site (i.e., depth independent of discharge) was measured as described by Lisle (1987), as the difference between channel depth at the riffle crest and the deepest point of the downstream pool. A temperature logger was placed at approximately medium depth in a haphazardly selected pool within each reach over the same 2‐week period to quantify relative variation in water temperature (0.1°C). Discharge (0.1 m^3^/s) was measured at the downstream and upstream end of each site with a Marsh McBirney Flo‐mate (Hach, Loveland Colorado) using the velocity‐area method (Gordon et al., 2004). Groundwater contribution was quantified following Zhou et al. (2018). The seepage contributions or losses (to the nearest 0.01 m^3^/s) were calculated by taking the difference between the downstream and upstream discharge calculations to estimate gaining or losing stream discharge for each site (Riggs, 1972).

Existing geospatial data were used to calculate reach‐scale covariates, which were applied to each nested site within a reach. We obtained the drainage area upstream of each reach (km^2^) from the National Hydrography dataset (Dewald, 2014, http://nhd.usgs.gov/). Drainage area is a primary structuring mechanism for fish distributions (Fausch et al., 2002; Schlosser, 1995). The percentage of agriculture upstream of our study reaches was calculated using the 2016 National Land Cover Dataset (Homer et al., 2015, http://mrlc.gov) to represent anthropogenic disturbance.

### 2.4 Occupancy modeling

We fit a single‐season, multispecies occupancy model using the hierarchical framework described by MacKenzie et al. (2002). Occupancy modeling uses the observation process (i.e., species encounter histories) to estimate occurrence probability while accounting for detection error. The latent occurrence state for species *i* at site *j* was treated as partially observed, with *z*_*ij*_ = 1 if the species was truly present and *z*_*ij*_ = 0 if the species was truly absent. Each *z*_*ij*_ followed a Bernoulli distribution with occurrence probability Ψ:

$$z_{\mathrm{ij}}\sim \text{Bernoulli}\;\left(\Psi_{\mathrm{ij}}\right)$$

The detection of species *i* at site *j* for survey *k* was conditional on both the true occurrence state and detection probability *p*, where encounter history *y*_*ijk*_ followed a Bernoulli distribution:

$$y_{\mathrm{ijk}}\sim \text{Bernoulli}\;\left({z_{\mathrm{ij}}}^{*}\;p_{\mathrm{ijk}}\right).$$

We modeled variation in Ψ and *p* using logistic regression beginning by fitting a full model containing all predictor variables (see Appendix A for details about covariate treatments model equations, and model selection). The detection equation contained the covariates percent coarse wood, percent coarse substrate, percent vegetation, water velocity, depth, water clarity, and water temperature. We included a quadratic term for depth to allow for a nonlinear relationship. We treated gear and ecoregion as indicator variables (0: seining, 1: snorkeling, and 0: Arbuckle Lift, 1: Ozark Highlands, respectively). We allowed species detection relationships to vary by both gear and ecoregion and gear detection relationships to vary with each covariate (i.e., gear‐covariate relationships were assumed to be the same among species). Species coefficients were modeled as deflections around the group mean hyperparameter governed by a probability distribution (Dorazio et al., 2006; Dorazio & Royle, 2005; Kéry and Royle, 2016). This allowed the relationships to be interpreted as though we fit a model for each species, as opposed to differences from a reference. The occurrence equation contained the reach‐scale covariates mean two‐week temperature, drainage area, and proportion of agricultural land use. The site level covariates were percent fine substrate, percent pool area, RPD, groundwater contribution, percent vegetation, and percent coarse wood. We also included three covariate interaction terms hypothesized to explain variation in species occurrence: water temperature * RPD (Peck et al., 2013), percent vegetation * RPD (Rozas & Odum, 1988; Savino & Stein, 1982), and percent vegetation * percent pool (Burr & Page, 1979; Hargrave & Johnson, 2003; Johnson & Hatch, 1991). We treated ecoregion and year as indicator variables (0:2018, 1:2019). We also included a stream reach grouping factor (i.e., “random intercept”, Gelman & Hill, 2006; Wagner et al., 2006) that varied by both species and ecoregion to account for unexplained variation in occurrence probability and spatial correlation of sites nested within a reach. Species coefficients were modeled as described for the detection equation, where we also allowed main effect relationships for covariates to vary between ecoregions. We simplified the model with a backward‐selection process based on the effect size and uncertainty of species relationships (see Appendix A). We fit models using the program JAGS (Plummer, 2003) called from the statistical software R (version 3.5.3; R Developments Core Team,  2019) with the package jagsUI (Kellner, 2021, see Appendix A for details).

For line plots presented in the Results, all detection and occurrence relationships are with other covariates held at mean levels and a mean year effect. Uncertainty in the plots reflects not only error around the slope, but also unexplained variation between ecoregions and years and among reaches. Any curvature in the lines is an artifact of the approximation of the probability on the logit scale to a value constrained between zero and one or the back transformation of the covariate from the natural‐log scale (or both). Relationships are linear on the modeled scales.

## 3 RESULTS

### 3.1 Fish sampling

We sampled 153 sites nested within 61 stream reaches in the Arbuckle Uplift and Ozark Highland ecoregions during 2018–2019 (Figure 1). Of the 153 sites, 42% (*n* = 64) were in the Arbuckle Uplift ecoregion, whereas 58% (*n* = 89) were in the Ozark Highlands ecoregion. During the two summers of sampling, we conducted 284 seining and 264 snorkel surveys across all sites. During the summer 2018, 69 sites were sampled: 26 sites in the Arbuckle Uplift and 43 in the Ozark Highlands. During summer 2019, we sampled 84 sites: 38 sites sampled in the Arbuckle Uplift and 46 sites sampled in the Ozark Highlands ecoregion.

The detection of our fishes differed by ecoregion, and some species were detected more often using one of the two gears. Least darter was encountered less than the other target species. Redspot chub was the most common species encountered in both ecoregions. Both age‐0 and age‐1+ smallmouth bass were encountered less in the Arbuckle Uplift than in the Ozark Highlands ecoregion. Least darter was detected at more sites in the Arbuckle Uplift ecoregion (*n* = 15) than in the Ozark Highlands ecoregion (*n* = 3), whereas redspot chub, and age‐0 and age‐1+ smallmouth bass were encountered at more than twice as many sites in the Ozark Highlands compared to the Arbuckle Uplift. Least darter was detected at about the same number of sites when seining (*n* = 18) or snorkeling (*n* = 24). Smallmouth bass, redspot chub, and southern redbelly dace were typically 2–3 times more likely to be detected by snorkeling than by seining, regardless of ecoregion. Redspot chub and smallmouth bass subadults were exceptions as the frequency of detections was similar in the Arbuckle Uplift regardless of gear used.

### 3.2 Physicochemical conditions

The physicochemical conditions associated with our surveys varied across sites but were similar between ecoregions and sample years (Table 1). Sites in both ecoregions had moderate amounts (~25%) of coarse substrates and coarse wood (~15%). Mean temperature (~23°C), water depth (~0.30 m), and mean water‐column velocity (~0.17 m/s) were similar across sites in each ecoregion. Percent vegetation and water clarity were more variable at sites in the Arbuckle Uplift ecoregion. Additionally, sites in the Arbuckle Uplift tended to have higher percentages of vegetation and lower water clarity than those in the Ozark Highlands.

Site‐level occurrence covariates varied across sites and between ecoregions. (Table 1). Water temperature, groundwater contribution, and proportion of agriculture were, on average, similar between ecoregions. The primary difference between sites in the two ecoregions was the percentage of fine substrates (Arbuckle Uplift, 39%; Ozark Highlands, 10%). Mean percent vegetation was higher in the Arbuckle Uplift (25%) than in the Ozark Highlands (15%). Lastly, RPD was greater in the Ozark Highlands (0.74 m) compared to the Arbuckle Uplift (0.54 m). Average physicochemical conditions between 2018 and 2019 were similar except mean water‐column velocities were slightly higher in 2019 (0.21 m/s) than in 2018 (0.11 m/s).

### 3.3 Occupancy modeling

**FIGURE 2:**
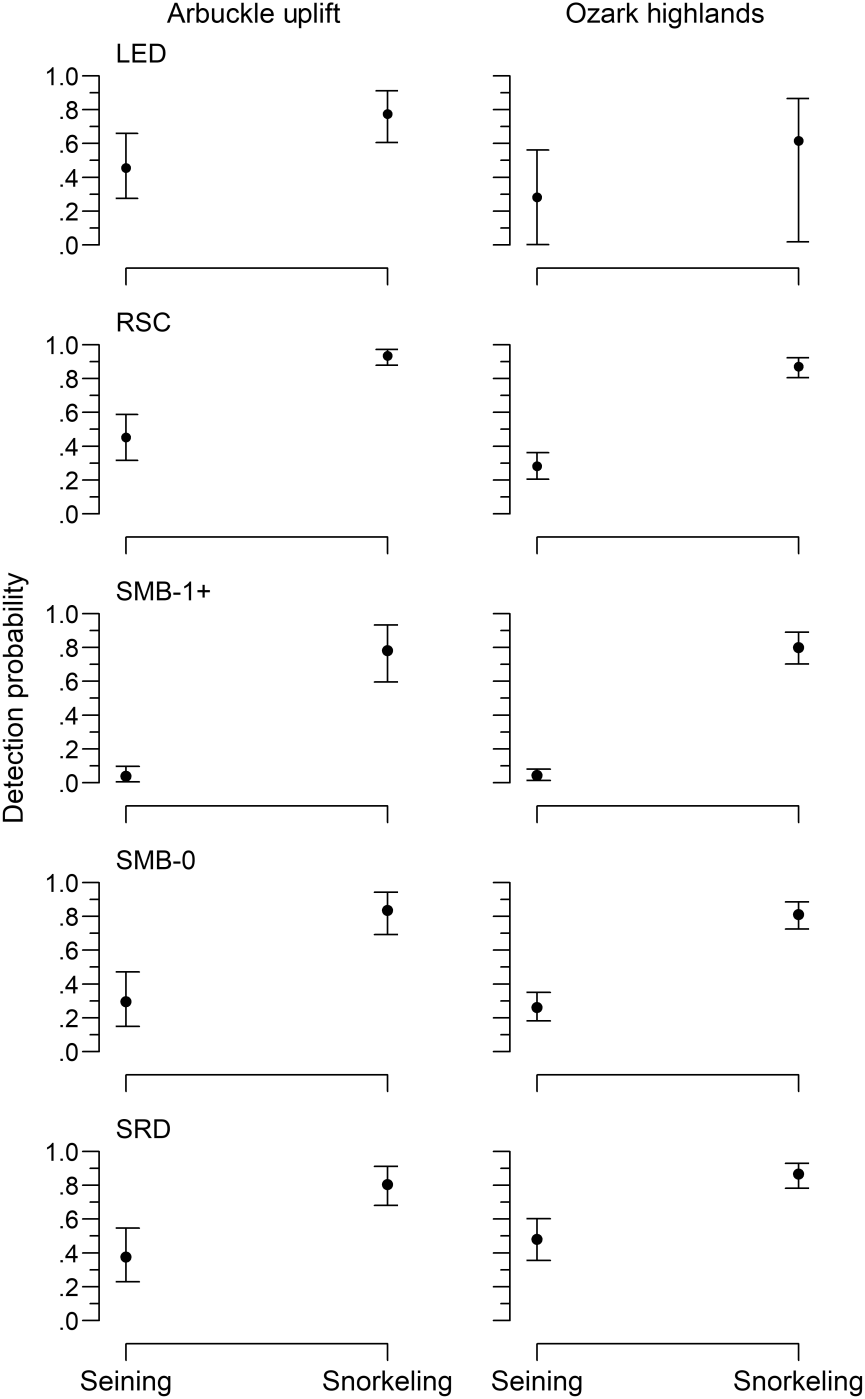
Detection probability among species by sampling gear type and ecoregion at mean levels of covariates. Circles are the mode (most likely value) from the posterior distribution and error bars represent endpoints of 95% highest density intervals. LED is least darter, RSC is redspot chub, SMB is smallmouth bass, and SRD is southern redbelly dace.

**FIGURE A1:**
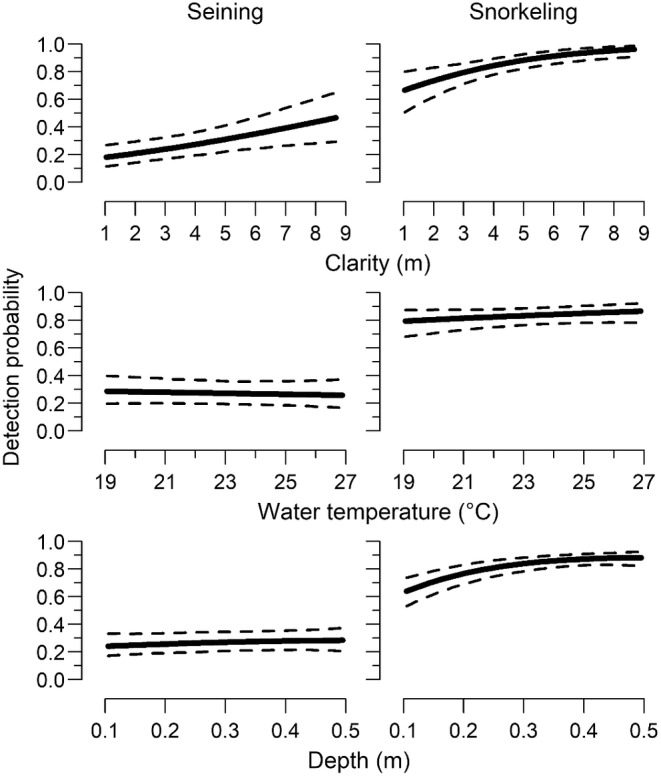
Relationship between fish detection probability and physicochemical covariates by gear type that met our criteria (see model selection in Appendix A). Dashed lines are 95% credible intervals.

Species detection probability (hereafter detection) varied both between sampling methods and in relation to covariates. On average, the detection was higher with snorkeling for all species in both ecoregions, though the HDIs overlapped for the least darter (Table A1, Figure 2). There were relationships between detection and the covariates of water velocity, water clarity, water temperature, and water depth (see Appendix A). Detection decreased with increasing water velocity, but the relationship did not differ between gear types. The positive detection relationship with water clarity was statistically different between gear types. However, the effect size was only slightly larger for seining (Table A1, Figure A1). Similarly, the detection across water temperatures differed statistically between seining and snorkeling, but this relationship was weak for both gear types (Table A1, Figure A1). Water depth had the most notable detection difference between gear types. Detection increased with water depth and reached an asymptote ~0.4 m (i.e., a quadratic relationship), but there was no relationship for seining (Table A1, Figure A1).

**FIGURE 3:**
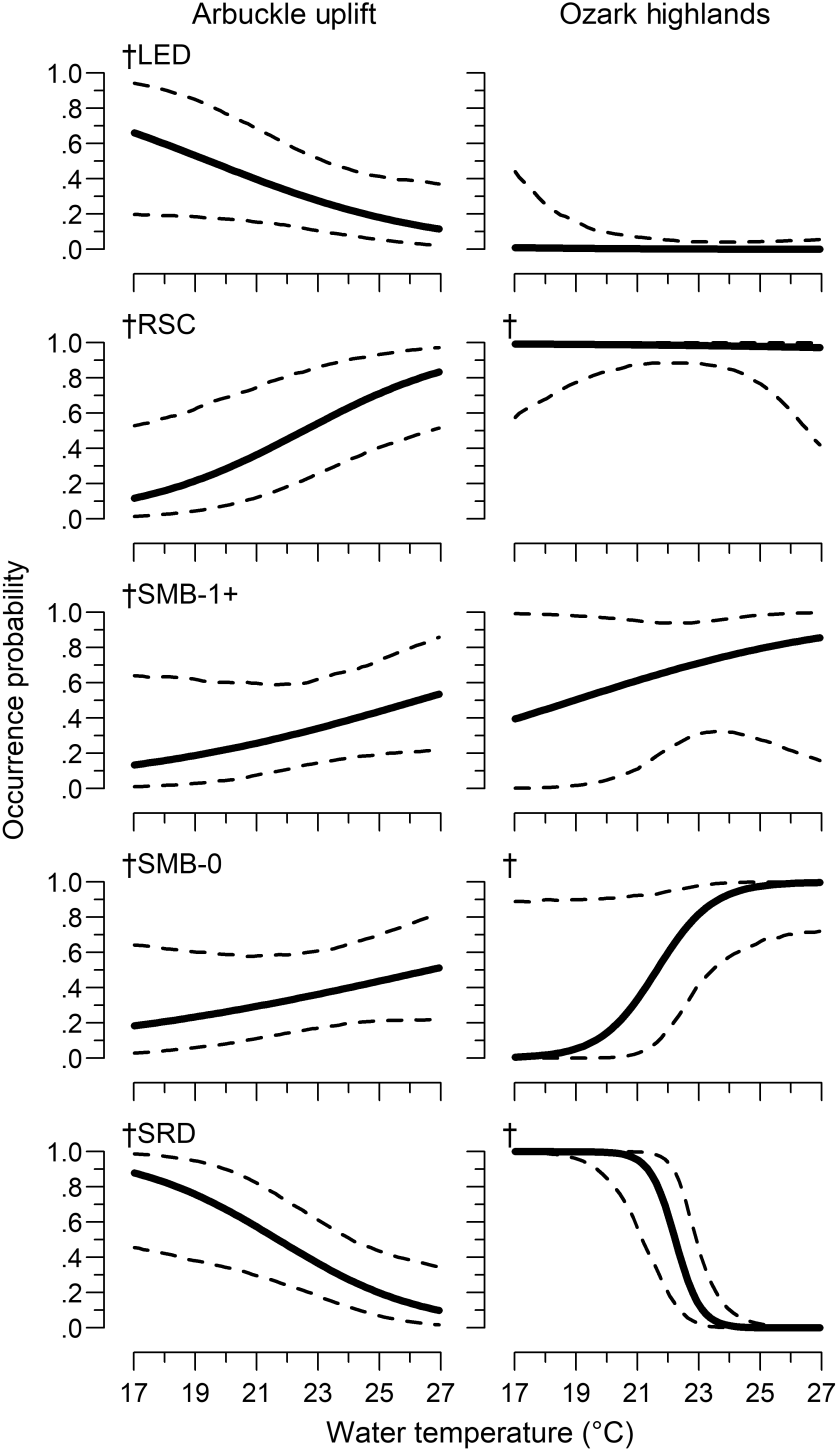
Relationship between occurrence probability and water temperature among species between ecoregions. Dashed lines are 95% credible intervals. LED is least darter, RSC is redspot chub, SMB is smallmouth bass, and SRD is southern redbelly dace. † for Arbuckle Uplift denotes relationships that met our criteria (see model selection in Appendix A). † for Ozark Highland denotes relationships that varied between ecoregions.

**FIGURE 4:**
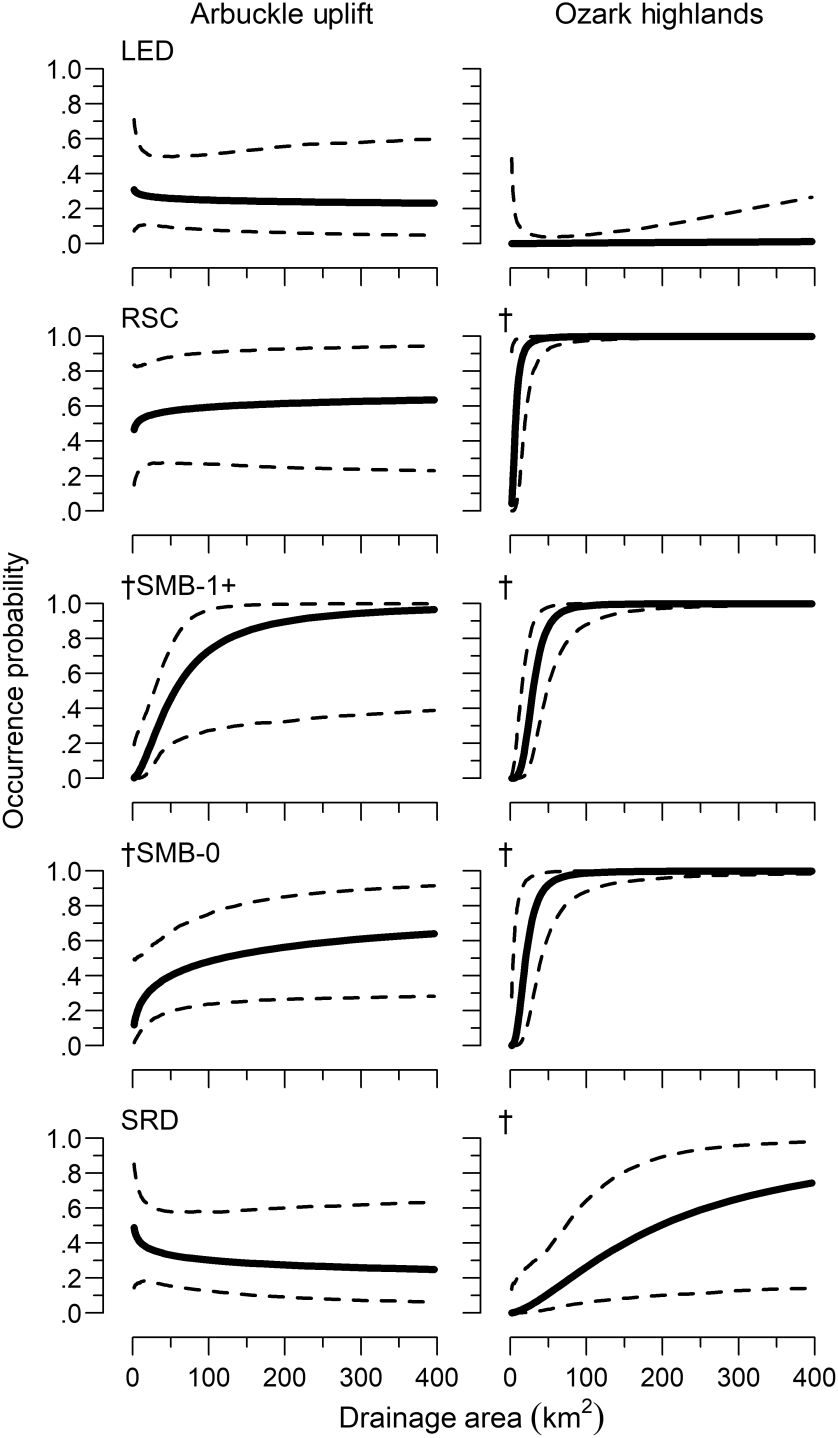
Relationship between occurrence probability and drainage area among species between ecoregions. Dashed lines are 95% credible intervals. LED is least darter, RSC is redspot chub, SMB is smallmouth bass, and SRD is southern redbelly dace. † for Arbuckle Uplift denotes relationships that met our criteria (see model selection in Appendix A). † for Ozark Highland denotes relationships that varied between ecoregions.

**FIGURE 5:**
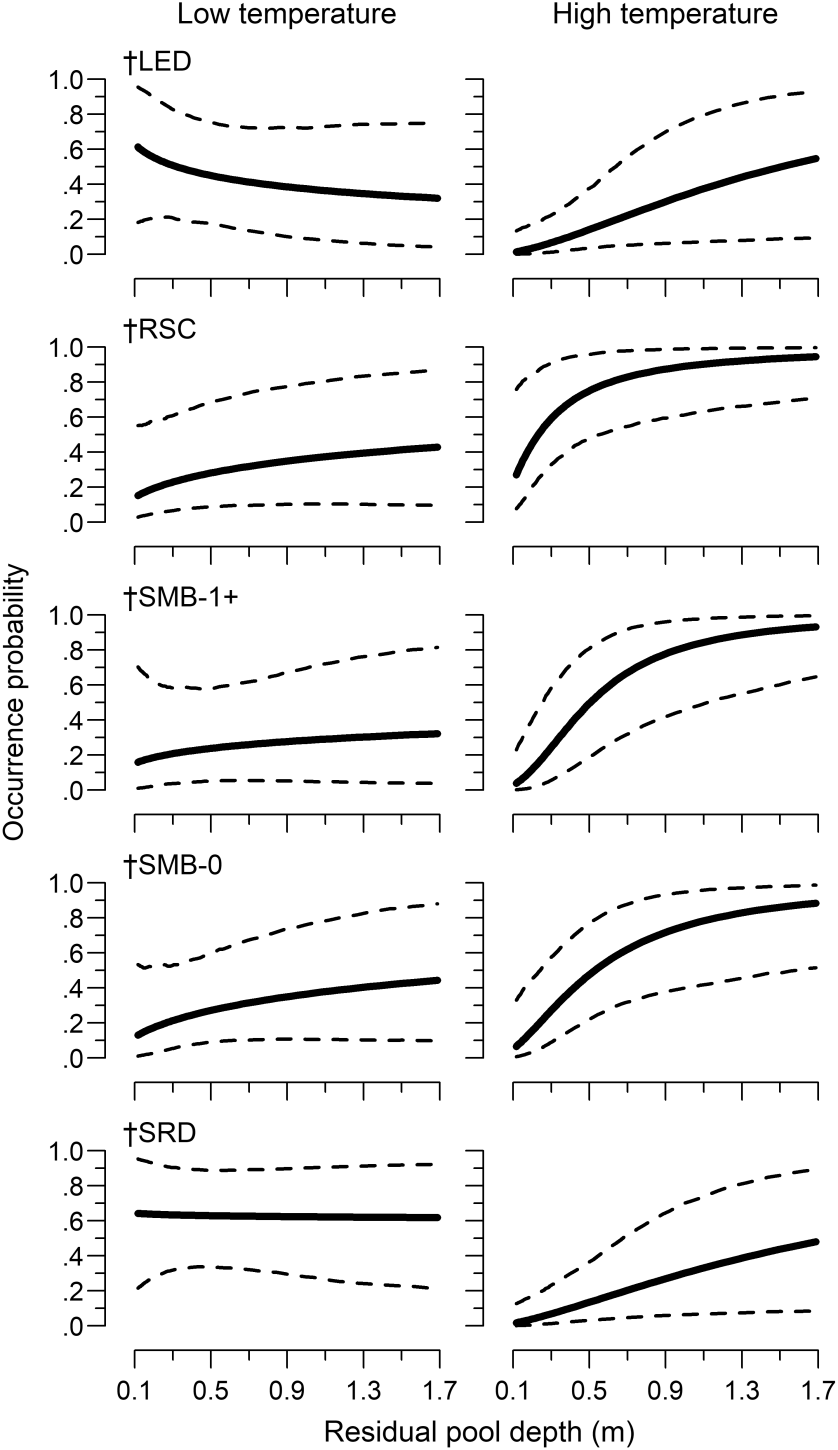
Relationship between occurrence probability and residual pool depth (RPD) among species at low and high‐water temperature (−1 and 1 SD, respectively). Dashed lines are 95% credible intervals. LED is least darter, RSC is redspot chub, SMB is smallmouth bass, and SRD is southern redbelly dace. † denotes water temperature‐RPD interaction terms that met our criteria (see model selection in Appendix A).

**FIGURE 6:**
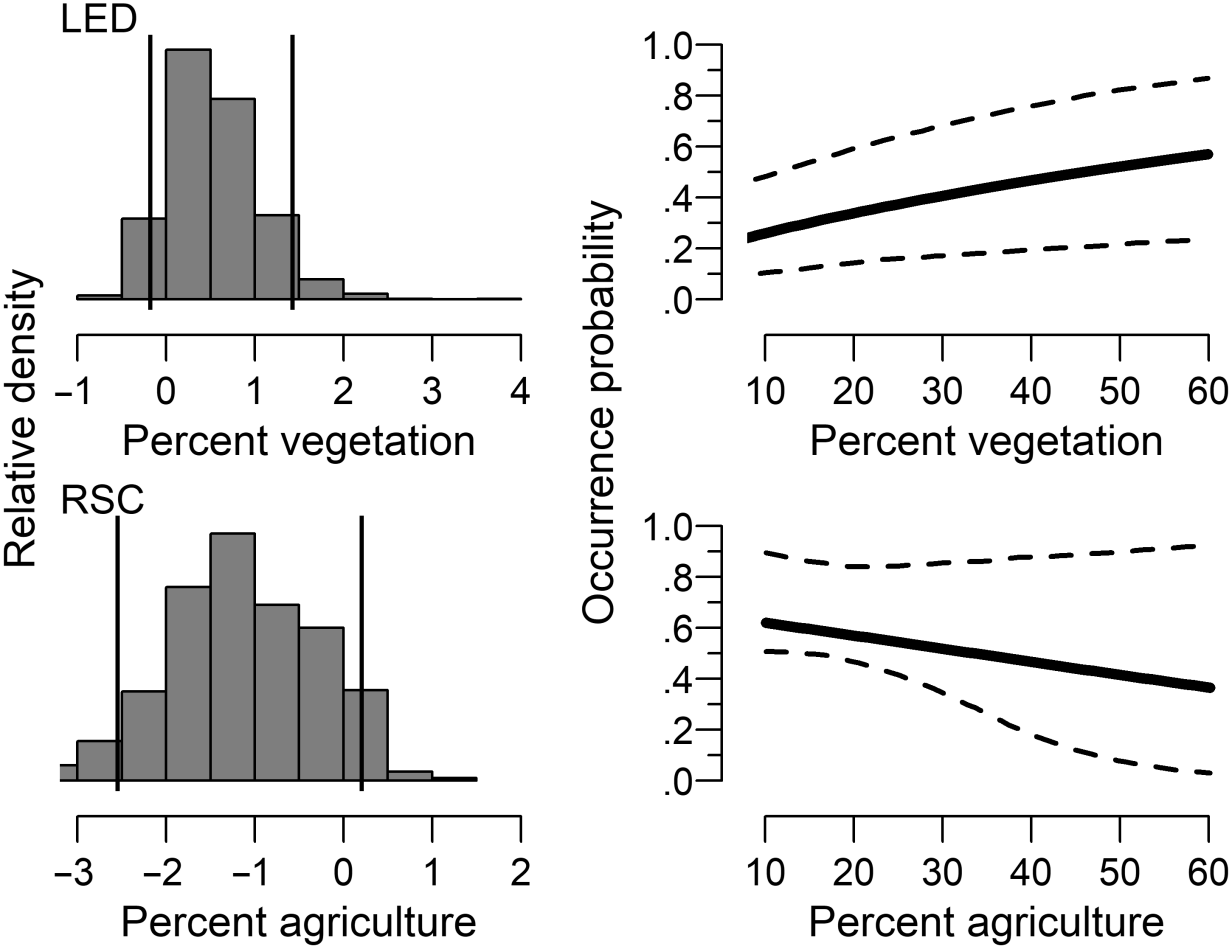
Relationship between occurrence probability and percent vegetation for least darter (LED, upper panels) and relationship between occurrence probability and percent agriculture for redspot chub (RSC, lower panels). The histograms in the left panels are a visual representation of the posterior distribution for the covariate slopes. The vertical lines approximate endpoints of 95% highest density intervals. Dashed lines in the right panels are 95% credible intervals.

**FIGURE A2:**
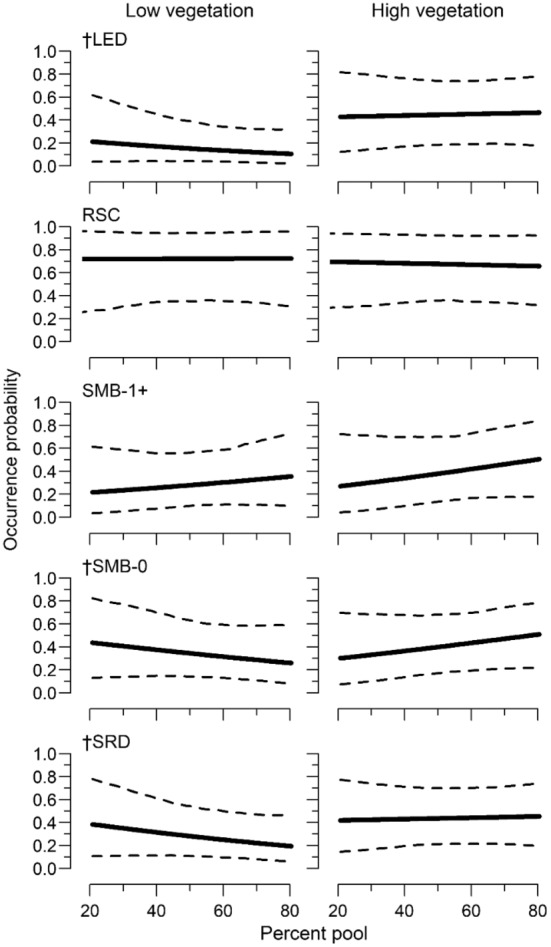
Relationship between occurrence probability and percent pool among fish species at low and high percent vegetation (−1 and 1 SD, respectively). Dashed lines are 95% credible intervals. LED is least darter, RSC is redspot chub, SMBa is age‐1 + smallmouth bass, SMBs is age‐0 smallmouth bass, and SRD is southern redbelly dace. † denotes important percent vegetation‐percent pool interaction terms that met our criteria (see model selection in Appendix A).

Species occurrence probability (hereafter occurrence) varied in relation to both site‐ and reach‐scale covariates, with differences between ecoregions. The final model retained site‐scale percent pool, percent vegetation, and RPD and reach‐scale water temperature, drainage area, and percent agriculture (see Appendix A for criteria). All species had a water temperature relationship, with some variation in occurrence between ecoregions. In the Arbuckle Uplift, occurrence decreased with increasing water temperature for least darter and southern redbelly dace but increased for redspot chub and both smallmouth bass life stages (Table A1, Figure 3). Least darter had little relationship with water temperature in the Ozark Highlands. The strength of the relationship was stronger in the Ozark Highlands for southern redbelly dace and age‐0 smallmouth bass. Redspot chub had no relationship with water temperature in the Ozark Highlands. Both redspot chub and southern redbelly dace had no occurrence relationship with the drainage area in the Ozark Highlands but had a strong positive relationship in the Arbuckle Uplift (Table A1, Figure 4). Occurrence increased with increasing drainage area in both ecoregions for both smallmouth bass age classes, with a stronger positive relationship in the Ozark Highlands. All species had a similar RPD relationship in both ecoregions that was dependent on water temperature (i.e., an interaction). The relationship was weak at lower water temperatures, but occurrence increased sharply with RPD at higher water temperatures (Table A1, Figure 5). There were single‐species occurrence relationships for percent vegetation and percent agriculture that also did not vary between ecoregions. Occurrence increased with increasing vegetation for least darter, while occurrence decreased with increasing agriculture for redspot chub (Figure 6). There was also statistical evidence of a percent vegetation and percent pool interaction. However, this resulted in only small changes in occurrence, with weak percent pool relationships at both low and high levels of vegetation (Figure A2).

## 4 DISCUSSION

We found that some physicochemical relationships for spring‐associated fishes differed by ecoregion, but many were similar. Although we can only speculate about the reasons, water temperature relationships differed in the two ecoregions for three of the five fishes we studied. We also found that smallmouth bass occurrence was relatively high in smaller and larger drainage areas of the Ozark Highlands, whereas bass was less likely to occur in smaller drainages of the Arbuckle Uplift. Alternatively, we found important ecological relationships were shared despite the separation in ecoregions. Most notably, we found an interaction between RPD and water temperature, regardless of ecoregion, where sites with deeper pools were more likely to be occupied by all fishes examined under higher water temperature conditions. In addition to identifying the ecological relationships of spring‐associated fishes, our findings have implications for stream‐fish conservation. In particular, instream habitat manipulations or protections may be useful strategies to counter increasing water temperatures due to climate change and other human perturbations.

Habitat degradation is a threat facing many aquatic species but has specific implications when considering future projected changes in stream water temperatures. Water temperatures in this region (MacDonald, 2010) and many others in North America (Poff et al., 2002) are rising, and projections indicate many aquatic species will make northward migrations (Ficke et al., 2007; Jackson & Mandrak, 2002; Shuter & Post, 1990). However, for narrow‐range endemic species with limits on the northward‐migration capacity (Carpenter et al., 1992), conservation efforts will likely focus on either moving populations (Galloway et al., 2016) or ensuring there is mitigation in place that will ensure persistence (Paukert et al., 2021). For example, least darter occupies catchments where downstream dispersal would be the only possible consideration without human intervention. Alternatively, managers might consider ensuring that deeper pool habitat is maintained, and groundwater connections persist (i.e., monitor groundwater withdrawals), so a natural thermal refugia is available to this and the other four species we examined. This is especially important for thermal conditions given the important role water temperature plays in the physiological and behavioral responses of fishes (Coutant, 1987) and a broader capacity as a resource fish use (Magnuson et al., 1979). Ensuring the continued persistence of key habitat patches will be important for the persistence of many riverine species.

Streams are inherently nested ecosystems; thus, it is important to understand patch dynamics and species distributions in a multiscale context (Fausch et al., 2002; Frissell et al., 1986; Gido et al., 2006). Relationships may emerge when examining environmental interactions across spatial scales. For example, water temperature was similar across adjacent sites but variable at the reach scale. We found that occurrence relationships with site‐scale pool characteristics (measured as RPD) were strongly dependent on reach‐scale water temperature for all focal species. This relationship is not surprising for larger‐bodied fishes (e.g., smallmouth bass, Dauwalter & Rahel, 2008). However, the association with deeper pools during warmer periods is interesting for smaller‐bodied species such as least darter and southern redbelly dace. The relationship between smaller‐bodied fishes and deeper pools is common in intermittent streams, where predation risk is low (Labbe & Fausch, 2000). However, understanding the dynamics between smaller and larger‐bodied species in larger streams where predation is a concern has not been well studied in warmwater streams. Coarser‐scale environmental characteristics also constrain and shape finer‐scale stream habitats (Frissell et al., 1986; Stevenson, 1997). Observed patterns in stream‐fish occurrence at a certain scale are associated with coarser‐scale factors (Mollenhauer et al., 2019). We found a higher occurrence probability with increasing drainage area for multiple species. Thus, all sites nested within a reach were more likely to be occupied in larger drainages, regardless of site‐scale characteristics.

Although fundamental to ecological research and conservation, multiscale studies are challenging and require appropriate study design and analytical approaches. Completely random or haphazard site selection does not alleviate the inevitable spatial correlation (pseudoreplication) when sampling nested systems (Fausch et al., 2002; Wagner et al., 2006). Further, sampling a single site within a coarser spatial scale (here one habitat complex per reach) does not allow for examining multiscale relationships (i.e., no replication). Rather, a multiscale design has intentional pseudoreplication (i.e., multiple habitat complexes with a reach), which can be dealt with via an applicable analysis. Multilevel (hierarchical) modeling can incorporate grouping factors to account for the lack of independence among nested observations (Gelman & Hill, 2006; Wagner et al., 2006). As we did here, covariates can also be included to identify coarser‐scale drivers of finer‐scale relationships (i.e., explain sources of spatial correlation). Multiple species can also be included in a single multilevel model but interpreted as a series of single‐species models. Due to multiple sources of error, multilevel models inherently (and appropriately) have increased uncertainty around estimates. However, a primary advantage is the ability to break a complex question into manageable pieces (i.e., isolate important relationships amidst the “noise”). Although Bayesian modeling is more “naturally” multilevel, the general approach is also readily applied in Frequentist statistics (i.e., random‐effects or mixed‐effect models; Gelman & Hill, 2006; Kéry and Royle, 2016).

Between‐ecoregion differences in habitat associations can be interesting and may play a key role in developing meaningful conservation and management strategies for fishes. For two fishes, there were relationships with water temperature in the Arbuckle Uplift but not the Ozark Highlands. For the least darter, the lack of relationship in the Ozark Highlands was likely related to the relatively few detections in that ecoregion, resulting in high uncertainty in the parameter estimate. However, the redspot chub had numerous detections and did not have a relationship between occurrence and water temperature in the Ozark Highlands (except for periods of warmer water temperatures). This may be related to biotic considerations, such as dispersal processes (Wisz et al., 2013), often not included in habitat models. Alternatively, if water temperatures do not exceed their critical maximums (i.e., 34.8°C for redspot chub, Farless & Brewer, 2017), there may be other context dependencies on this relationship. For example, sizes of redspot chub in the Ozark Highlands exhibit two feeding niche patterns (Rodger & Starks, 2021), which may relate to changing habitat patterns based on food resources available. This may be one of several reasons others show ecological relationships in some studies that are not mirrored by others. Unfortunately, studies examining one or a few rivers cannot uncover these relationships. Relationships with drainage area also differed by ecoregion for four of the five species we examined (i.e., all but least darter). Interestingly, the four species tend to be more common in the Ozark Highlands, and we see their occupancy approach a high level for all species except southern redbelly dace. One possibility is that density dependence affects how some species use their habitat. This phenomenon is documented for salmonid life stages (e.g., juvenile brown trout Lobón‐Cerviá, 2006; Lobón‐Cerviá & Mortensen, 2006). For smallmouth bass, however, the population inhabiting the Arbuckle Uplift is non‐native (Starks & Rodger, 2020), whereas a different native strain occurs in the Ozarks Highlands (Brewer & Orth, 2015).

Differences in the recent taxonomic divisions of smallmouth bass may also relate to some differences observed in occupancy relationships. Smallmouth bass in the Ozark Highlands of OK, southeast KS, and southwest MO were once considered a subspecies of smallmouth bass (Hubbs & Bailey, 1940). More recently, it has been suggested that the subspecies be elevated to the species level (Kim et al., 2022). Research in the Ozark Highlands shows the Neosho bass (*M. dolomieu velox*) tends to use smaller streams more so than the nominal species (Miller & Brewer, 2020; Taylor et al., 2018), which was introduced to the Arbuckle Uplift. Additionally, small streams adjacent to larger rivers are important rearing areas for the Neosho bass (Miller & Brewer, 2020). Differences in their ecology may be related to underlying differences in evolution in the Ozark Highlands. This is important as closely related species are often assumed to have similar ecological needs, which is not the case for many species and hinders our ability to designate surrogate species.

Although we outline some interesting between and within ecoregion relationships, examination of network position of physicochemical parameters would be an area where future research might focus. Smaller streams, for example, can be important rearing habitats even for top‐level predators (Meyer et al., 2007; Rosenfeld et al., 2002), and spatial proximity can be a driving factor (Smith & Kraft, 2005). For example, Miller and Brewer (2020) found that smaller streams of the Ozark Highlands adjacent to larger streams support relatively large populations of young‐of‐year Neosho bass. The adjacent larger streams provide heterogeneous habitats, including thermal patchiness (Arrigoni et al., 2008; Westhoff & Paukert, 2014), diverse foraging opportunities (Sabo et al., 1996), and refuge from disturbance, predation, and density‐dependent effects (Letcher et al., 2015; Lukas & Orth, 1995). Consequently, including network position in future studies would provide insight into the importance of habitat patches across space. Conservation efforts focused on certain portions of the landscape (i.e., springs) that are particularly ecologically meaningful may also help focus our use of limited conservation dollars.

Snorkeling in relatively clear warmwater streams is often overlooked as a sampling strategy; however, it has several advantages over more traditional sampling gears, depending on the study goal. We show an increase in detection probability while snorkeling as water depth, water clarity, and water temperature increase. Surprisingly, even at lower visibilities (~1 m), snorkeling had higher detection than seining for our target species. Others have also found that snorkeling performs as well as the comparative method (Albanese et al., 2011; Brewer & Ellersieck, 2011; Moore et al., 2017). Snorkeling offers many advantages over seining for stream fishes, such as no handling stress and less physical habitat disturbance. These advantages are especially true when studying threatened or endangered species. Snorkeling also limits harm to non‐target species and taxa. Snorkeling has also been used for reliable abundance and size‐structure estimates compared to other methods (Mullner et al., 1998; Wildman & Neumann, 2003). As with any sampling method, snorkeling may not be appropriate for all fishes. For example, detection is very low for species with cryptic coloration (Macnaughton et al., 2015) and a high affinity for cover (Mollenhauer & Brewer, 2018). Nevertheless, future sampling efforts in relatively clear warmwater streams would benefit from the consideration of snorkeling as a primary sampling method for many species after an initial evaluation. Snorkeling is a method that would add to the repertoire of standard sampling methods for warmwater North American fishes.

Habitat alterations due to landscape changes not captured by agricultural disturbance may affect species in both ecoregions (Christenson et al., 2011; Seilheimer & Fisher, 2010). We found a negative relationship between redspot chub occurrence probability and the amount of upstream agriculture, regardless of ecoregion (Figure 6). The apparent decrease in suitable habitats for spring‐associated species at several of the known historical locations could be due to a myriad of factors, including altered flow regimes (Lynch et al., 2018; Poff et al., 1997), climate change (Hu et al., 2005), or introduced species (Rahel et al., 2008). Least darter and southern redbelly dace were more widespread in the Ozark Highlands in historical collections before 1970. The Ozark Highland sites had higher percentages of agriculture, and human landscape activities can intensify patchy distributions at range edges (Sagarin et al., 2006). Our agriculture coefficient did not include other catchment‐level disturbances and may co‐vary (i.e., land use totals to 100%). Future research would benefit from directly examining the mechanisms associated with landscape perturbations (e.g., sedimentation) to determine the specific stressor.

Multiscale studies of lotic warmwater species are important for developing meaningful conservation plans (Labbe & Fausch, 2000; Torgersen et al., 2006; Wang et al., 2001). As climate change continues to alter the landscape, conservation strategies will be especially important for those species with lower thermal tolerances associated with springs or other groundwater features. Spring‐associated species rely on hyporheic flows because the constant water temperature minimizes extreme fluctuations, creates thermal refugia, and helps maintain baseflows (Bergey et al., 2008; Matthews et al., 1985; Peterson & Rabeni, 1996; Schaefer et al., 2003; Torgersen et al., 1999). Interestingly, we show how all our warmer target species occupied deeper pools during periods of warmer‐water conditions, indicating that consideration for maintaining appropriate channel morphology may benefit a variety of warmwater fishes. This may be especially important given the sheer number of warmwater fishes that have been petitioned for listing within the United States, especially the more diverse areas of the southeast (https://ecos.fws.gov/ecp/report/table/petitions‐received.html, Accessed September 15, 2023).

Managers are increasingly concerned about the persistence of warmwater fishes; our results indicate some possibilities for stream temperature mitigation, and we identify stressors that are shared across both ecoregions. Efforts to identify the mechanistic underpinning of these ecological relationships would be helpful to discern if some of the different ecoregion relationships represent warning signals caused by physicochemical stressors. For example, do the shifting temperature relationships in the two ecoregions signal concerns related to survival and growth, or is this a plastic response to shifting prey resources (and therefore a tradeoff with water temperature conditions)? Our results also indicate that channel mitigation to maintain reaches with deeper pool habitats may be an important strategy for maintaining thermal refugia during warmer water temperatures. For example, preventing gravel removal is a management strategy aimed at preventing the widening and shallowing of stream channels. Moreover, some agricultural and urban land uses cause the widening and shallowing of stream channels (Kayitesi et al., 2022; Konrad & Booth, 2005), particularly when the riparian corridor has not been properly maintained (Lovell & Sullivan, 2006; Naiman & Decamps, 1997). Although we recognize that species are plastic and that may relate to resilience to climate change (Seebacher et al., 2015), it is unclear whether thermally sensitive warmwater fishes can adapt their thermal tolerances at a rate that keeps up with warming trends. It will become increasingly important to identify strategies that will provide apparent refugia if the goal is to conserve many of our warmwater fishes.

## AUTHOR CONTRIBUTIONS

**Dusty Swedberg:** Conceptualization (equal); data curation (lead); formal analysis (equal); funding acquisition (supporting); investigation (equal); methodology (lead); project administration (supporting); writing – original draft (lead); writing – review and editing (supporting). **Robert Mollenhauer:** Conceptualization (supporting); data curation (supporting); formal analysis (equal); funding acquisition (supporting); investigation (equal); methodology (equal); project administration (supporting); visualization (supporting); writing – review and editing (equal). **Shannon K. Brewer:** Conceptualization (lead); data curation (supporting); formal analysis (supporting); funding acquisition (lead); investigation (equal); methodology (equal); project administration (lead); supervision (lead); writing – original draft (equal); writing – review and editing (lead).

## Data Availability

Data from this study are available at https://datadryad.org/stash/share/WjyFq4‐gSU8c7jUgtGN741DIsvxcbi8ZOemxEwgFciQ (Dusty Swedberg, swedberg@illinois.edu).

## APPENDIX A

### A.1 Covariate transformations and pairwise correlations

Prior to fitting models, we made data transformations to improve linearity and checked multicollinearity among covariates. We natural‐log transformed water velocity, discharge, drainage area, residual pool depth, and site area due to right‐skewed distributions. We improved symmetry for percent vegetation, percent coarse wood, and percent fine substrate using a square‐root transformation. We used Pearson's pairwise correlation coefficient (*r*) to examine multicollinearity. There were no strong correlations between our detection covariates (|*r*| ≤ .26). We removed discharge and total area from our occurrence covariate set because they were strongly positively correlated with drainage area (|*r*| ≥ .89). The final occurrence covariate set maintained |*r*| ≤ .54.

### A.2 Model equations

We write the detection equation as:

$$\begin{array}{cc} \text{logit}\left(p_{\mathrm{ijk}}\right) & =\alpha_{i}+\alpha_{\text{1i}}E_{j}+\alpha_{2i}G_{\mathrm{jk}}+\sum_{n=1}^{7}\beta_{n}X_{\mathrm{njk}}\\ & +\beta_{8}X_{7\mathrm{jk}}^{2}+{\sum_{b=9}^{15}\sum }_{n=1}^{7}\beta_{b}X_{\mathrm{njk}}G_{\mathrm{jk}}+\beta_{16}X_{7\mathrm{jk}}^{2}G_{\mathrm{jk}.} \end{array}$$

for *i* = 5, for *j* = 153, for *k* = 1, 2, … *K*

$$\alpha \sim \text{Gaussian}\left(\mu_{\alpha },\sigma_{\alpha }^{2}\right),\alpha_{1}\sim \text{Gaussian}\left(\mu_{\text{α1}},\sigma_{\alpha 1}^{2}\right),\alpha_{2}\sim \text{Gaussian}\left(\mu_{\text{α2}},\sigma_{\alpha 2}^{2}\right),$$

where α is the species intercept, α_1_ is the ecoregion *E* coefficient, α_2_ is the gear *G* coefficient, β_1_–β_7_ are main effect coefficients for associated covariates mean velocity *X*_1_, percent coarse wood *X*_2_, percent coarse substrate *X*_3_, clarity *X*_4_, temperature *X*_5_, percent vegetation *X*_6_, and water depth *X*_7_ (main effect is the coefficient for the first‐order quadratic term), β_8_ is the coefficient second‐order quadratic water depth term, β_9_–β_15_ are gear interaction coefficients for associated covariates *X*_1_–*X*_7,_ β_16_ is the gear interaction coefficient for the second‐order quadratic water depth term, υ is a normality parameter, μ is the group mean hyperparameter, and σ^2^ is variance. We used a *t*‐distribution, rather than a normal distribution, for the reach grouping factor to account for heavy tails and improve model fit (Kruschke 2013; Lee and Thompson 2008).

We write the occurrence equation as:

$$\begin{array}{cc} \text{logit}\left(\Psi_{\mathrm{ijm}}\right) & =\alpha_{i}+\alpha_{\text{1i}}E_{j}+\alpha_{2i}T_{j}+\sum_{n=1}^{9}\beta_{\mathrm{ni}}X_{\mathrm{nj}}\\ & +\beta_{10i}X_{1j}\;X_{\text{8j}}+\beta_{11i}X_{6j}\;X_{\text{8j}}+\beta_{12i}X_{5j}\;X_{\text{6j}}+\sum_{n=13}^{22}\beta_{\mathrm{ni}}E_{j}X_{\mathrm{nj}}\;\\ & +\tau_{im},\;\text{for}\;i=5,\text{for}\;j=153,\text{for}\;m=61, \end{array}$$

$$\begin{array}{c} \alpha \sim \text{Gaussian}\left(\mu_{\alpha },\sigma_{\alpha }^{2}\right),\alpha_{1}\sim \text{Gaussian}\left(\mu_{\text{α1}},\sigma_{\alpha 1}^{2}\right),\\ \alpha_{2}\sim \text{Gaussian}\left(\mu_{\text{α2}},\sigma_{\alpha 2}^{2}\right),\\ \beta_{1–22}\sim \text{Gaussian}\left(\mu_{\beta 1–13,}\;\sigma_{\beta 1–13}^{2}\right),\tau \sim t\left(0,{\sigma_{\tau }^{2}}_{,}\;\upsilon_{\tau }\right), \end{array}$$

where α is the species intercept, α_1_ is the ecoregion *E* coefficient, α_2_ is the year *T* coefficient, β_1_–β_9_ are main effects coefficients or slopes for associated covariates mean temperature *X*_1_, percent coarse wood *X*_2_, percent fine substrate *X*_3_, percent agriculture *X*_4_, percent pool *X*_5_, percent vegetation *X*_6_, groundwater contribution *X*_7_, RPD *X*_8_, and drainage area *X*_9_, β_10_ is the mean temperature * RPD interaction coefficient, β_11_ is the percent vegetation * RPD interaction coefficient, β_12_ is the proportion pool * percent vegetation interaction coefficient, β_1_–β_9_ are coefficients for the ecoregion * covariate interaction terms, τ is the reach grouping factor, μ is the group mean hyperparameter, and σ is variance.

### A.3 Model selection

We simplified the occupancy model by removing covariates with a backward‐selection process. We began by removing gear‐detection covariate interaction terms if the absolute value of the mode (most likely value) in the posterior distribution was less than both endpoints of the 95% highest density interval (HDI) for the coefficient. This approach alleviated coefficients being removed for HDIs that slightly overlapped zero using a Frequentist‐like cutoff rule and shifted the focus from significance to effect size and uncertainty. Unlike confidence intervals that only provide endpoints, posterior distributions, and associated HDIs are direct expressions of effect size and uncertainty that can be expressed visually as a probability distribution (Kruschke and Liddell 2018). For retained terms, there was support for either a positive or negative relationship with the uncertainty primarily on the true effect size (see Figure 6 in the Results for an example). We refitted the model and used the same criteria to remove or retain the main effects without a gear interaction. A covariate interaction or main effect was retained if there was support of a relationship for at least one species. We considered retained ecoregion and gear interactions variable (e.g., the relationship between clarity and detection varied by gear type) and covariate interactions a dependency of one covariate relationship on levels of the other (e.g., the relationship between occurrence and residual pool depth was dependent on water temperature). We used the same process and criteria to simplify the occurrence component of the model, starting with ecoregion‐covariate interactions. We present occurrence and detection relationships visually in the Results, with the full output provided in the Appendix A (Table A1).

### A.4 JAGS specifications and diagnostics

Posterior distributions for coefficients were estimated with Markov chain Monte Carlo (MCMC) methods. We used truncated vague normal priors (Kéry and Shaub 2012; Kéry and Royle 2016) for group‐mean hyperparameters, vague gamma priors for associated standard deviations (Plummer 2003), and a shifted exponential prior for the occurrence reach grouping factor normality parameter (Kruschke 2013). Adequate MCMC convergence (chain mixing) was achieved using four chains of 50,000 iterations each ran in parallel after a 25,000‐iteration burn‐in phase (thinning = 100). We considered convergence a potential scale reduction factor < 1.05 (Brooks and Gelman 1998). We calculated HDIs using the R package HDInterval (Meredith and Kruschke 2022).

We examined model fit using a posterior predictive check (Conn et al. 2018; Kéry and Royle 2016). We simulated expected *y*_*js*_ under model parameters to compare discrepancies with observed *y*_*js*_ and calculated a Bayesian *p*‐value. A Bayesian *p*‐value near 0.5 suggests adequate fit and extreme values (i.e., <0.05 or >0.95) indicate a lack of fit. We also examined a plot of the discrepancy (i.e., residual error) of simulated data versus the discrepancy of observed data. A random scattering of values around the diagonal equality line supports a good fit. The Bayesian *p*‐value was 0.57 and the visual assessment confirmed adequate fit.

**TABLE A1:** Multispecies occupancy model output on the logit scale as the mode (most likely value) with a 95% highest density interval (HDI).

Coefficient	Mode (90% HDI)
Occurrence probability	
LED – Arbuckle Uplift, 2018	−0.11 (−1.73, 1.56)
LED – Ozark Highlands	−6.32 (−13.30, 2.46)
LED – 2019	−1.60 (−3.29, 0.12)
LED – water temperature (Arbuckle Uplift)^a^	−0.81 (−2.00, 0.25)
LED – water temperature (Ozark Highlands)	−0.45 (−4.34, 3.59)
LED – drainage area (Arbuckle Uplift)	0.26 (−0.85, 1.29)
LED – drainage area (Ozark Highlands)	2.20 (−2.47, 5.30)
LED – percent agriculture	0.05 (−1.32, 1.40)
LED – percent pool	−0.14 (−1.03, 0.57)
LED – RPD	0.56 (−0.76, 1.41)
LED – percent vegetation^a^	0.48 (−0.45, 1.47)
LED – RPD * water temperature^a^	0.94 (0.12, 2.29)
LED – percent pool * percent vegetation^a^	0.38 (−0.31, 1.18)
LED – reach SD	1.24 (0.01, 3.50)
RSC – Arbuckle Uplift, 2018	0.71 (−0.69, 2.74)
RSC – Ozark Highlands	4.53 (1.02, 8.72)
RSC – 2019	−1.55 (−2.96, −0.08)
RSC – water temperature (Arbuckle Uplift)^a^	1.06 (0.07, 2.08)
RSC – water temperature (Ozark Highlands)^a^	−1.75 (−5.50, 1.13)
RSC – drainage area (Arbuckle Uplift)	0.39 (−0.49, 1.39)
RSC – drainage area (Ozark Highlands)^a^	3.31 (0.19, 6.44)
RSC – percent agriculture^a^	−1.16 (−2.69, 0.41)
RSC – percent pool	−0.04 (−0.67, 0.73)
RSC – RPD^a^	0.81 (0.18, 1.64)
RSC – percent vegetation	−0.11 (−0.73, 0.54)
RSC – RPD * water temperature^a^	0.34 (−0.25, 1.04)
RSC – percent pool * percent vegetation	−0.01 (−0.75, 0.54)
RSC – reach SD	2.11 (0.66, 0.44)
SMB‐1+ − Arbuckle Uplift, 2018	0.18 (−1.37, 2.08)
SMB‐1+ − Ozark Highlands	1.59 (−1.36, 4.12)
SMB‐1+ − 2019	−1.50 (−3.03, 0.05)
SMB‐1+ − water temperature (Arbuckle Uplift)^a^	0.69 (−0.67, 2.08)
SMB‐1+ − water temperature (Ozark Highlands)	−0.34 (−3.25, 3.14)
SMB‐1+ − drainage area (Arbuckle Uplift)^a^	2.55 (0.27, 6.27)
SMB‐1+ − drainage area (Ozark Highlands)^a^	2.98 (0.12, 5.68)
SMB‐1+ − percent agriculture	−0.31 (−1.68, 1.02)
SMB‐1+ − percent pool	0.29 (−0.49, 1.58)
SMB‐1+ − RPD^a^	1.06 (0.13, 2.38)
SMB‐1+ − percent vegetation	−0.01 (−0.94, 0.81)
SMB‐1+ − RPD * water temperature^a^	0.79 (−0.05, 1.97)
SMB‐1+ − percent pool * percent vegetation	0.21 (−0.58, 1.04)
SMB‐1+ − reach SD	2.10 (0.59, 4.44)
SMB‐0 – Arbuckle Uplift, 2018	0.28 (−1.08, 1.55)
SMB‐0 – Ozark Highlands	2.73 (0.03, 5.63)
SMB‐0 – 2019	−1.70 (−3.43, −0.16)
SMB‐0 – water temperature (Arbuckle Uplift)^a^	0.59 (−0.47, 1.60)
SMB‐0 – water temperature (Ozark Highlands)^a^	2.30 (−1.55, 7.20)
SMB‐0 – drainage area (Arbuckle Uplift)^a^	0.82 (−0.17, 1.90)
SMB‐0 – drainage area (Ozark Highlands)^a^	3.40 (0.38, 6.37)
SMB‐0 – percent agriculture	0.52 (−0.76, 2.26)
SMB‐0 – percent pool	−0.08 (−0.84, 0.78)
SMB‐0 – RPD^a^	1.01 (0.16, 2.10)
SMB‐0 – percent vegetation	−0.13 (−0.95, 0.62)
SMB‐0 – RPD * water temperature^a^	0.53 (−0.48, 1.21)
SMB‐0 – percent pool * percent vegetation^a^	0.44 (−0.15, 1.24)
SMB‐0 – reach SD	2.51 (0.72, 4.90)
SRD – Arbuckle Uplift, 2018	0.13 (−1.39, 1.53)
SRD – Ozark Highlands	−2.11 (−5.45, 0.63)
SRD – 2019	−1.44 (−2.92, 0.14)
SRD – water temperature (Arbuckle Uplift)^a^	−1.32 (−2.89, −0.08)
SRD – water temperature (Ozark Highlands)^a^	−6.35 (−11.80, −0.36)
SRD – drainage area (Arbuckle Uplift)	−0.03 (−1.16, 1.05)
SRD – drainage area (Ozark Highlands)^a^	2.93 (0.18, 5.36)
SRD – percent agriculture	0.18 (−1.09, 1.55)
SRD – percent pool	−0.24 (−1.20, 0.49)
SRD – RPD^a^	0.61 (−0.39, 1.49)
SRD – percent vegetation	0.07 (−0.61, 0.88)
SRD – RPD * water temperature^a^	0.66 (−0.09, 1.92)
SRD – percent pool * percent vegetation^a^	0.33 (−0.26, 0.93)
SRD – reach SD	1.84 (0.44, 3.92)
Detection probability	
LED – Arbuckle Uplift, seining	−0.13 (−0.86, 0.77)
LED – Ozark Highlands	−0.73 (−4.69, 0.43)
LED – snorkeling	1.35 (0.29, 2.57)
RSC – Arbuckle Uplift, seining	−0.21 (−0.74, 0.38)
RSC – Ozark Highlands	−0.74 (−1.39, −0.04)
RSC – snorkeling	2.84 (2.23, 3.42)
SMB‐1+ − Arbuckle Uplift, seining	−3.21 (−4.48, −2.07)
SMB‐1+ − Ozark Highlands	0.09 (−0.82, 1.15)
SMB‐1+ − snorkeling	4.52 (3.58, 5.72)
SMB‐0 – Arbuckle Uplift, seining	−0.87 (−1.61, 0.03)
SMB‐0 – Ozark Highlands	−0.17 (−0.95, 0.70)
SMB‐0 – snorkeling	2.51 (1.87, 3.20)
SRD – Arbuckle Uplift, seining	−0.47 (−1.13, 0.29)
SRD – Ozark Highlands	0.40 (−0.32, 1.11)
SRD – snorkeling	1.93 (1.25, 2.71)
Velocity (seining and snorkeling)^a^	−0.13 (−0.28, 0.01)
Clarity (seining)^a^	0.34 (0.10, 0.54)
Clarity (snorkeling)^a^	0.27 (−0.09, 0.65)
Water temperature (seining)	−0.07 (−0.31, 0.16)
Water temperature (snorkeling)^a^	0.25 (−0.16, 0.60)
Depth (seining)	0.08 (−0.12, 0.30)
Depth^2^ (seining)	−0.03 (−0.20, 0.13)
Depth (snorkeling)^a^	0.47 (0.15, 0.79)
Depth^2^ (snorkeling)^a^	−0.19 (−0.38, 0.12)

*Note*: Coefficients for occurrence covariate relationships with the Ozark Highlands ecoregion represent the difference from the Arbuckle Uplift ecoregion. Coefficients for detection covariate relationships with snorkeling represent the difference from seining.

Abbreviations: LED, least darter; RPD, residual pool depth; RSC, redspot chub; SD, standard deviation; SMB, smallmouth bass; SRD, southern redbelly dace.

^a^ Variable covariate relationships (see model selection for criteria).

Any use of trade, firm, or product names is for descriptive purposes only and does not imply endorsement by the U.S. Government.
